# Association of artificial intelligence-powered and manual quantification of programmed death-ligand 1 (PD-L1) expression with outcomes in patients treated with nivolumab ± ipilimumab

**DOI:** 10.1038/s41379-022-01119-2

**Published:** 2022-07-15

**Authors:** Vipul Baxi, George Lee, Chunzhe Duan, Dimple Pandya, Daniel N. Cohen, Robin Edwards, Han Chang, Jun Li, Hunter Elliott, Harsha Pokkalla, Benjamin Glass, Nishant Agrawal, Abhik Lahiri, Dayong Wang, Aditya Khosla, Ilan Wapinski, Andrew Beck, Michael Montalto

**Affiliations:** 1 Bristol Myers Squibb, Princeton, NJ USA; 2 PathAI, Boston, MA USA

**Keywords:** Immunotherapy, Cancer imaging, Tumour biomarkers, Diagnostic markers, Cancer immunotherapy

## Abstract

Assessment of programmed death ligand 1 (PD-L1) expression by immunohistochemistry (IHC) has emerged as an important predictive biomarker across multiple tumor types. However, manual quantitation of PD-L1 positivity can be difficult and leads to substantial inter-observer variability. Although the development of artificial intelligence (AI) algorithms may mitigate some of the challenges associated with manual assessment and improve the accuracy of PD-L1 expression scoring, use of AI-based approaches to oncology biomarker scoring and drug development has been sparse, primarily due to the lack of large-scale clinical validation studies across multiple cohorts and tumor types. We developed AI-powered algorithms to evaluate PD-L1 expression on tumor cells by IHC and compared it with manual IHC scoring in urothelial carcinoma, non-small cell lung cancer, melanoma, and squamous cell carcinoma of the head and neck (prospectively determined during the phase II and III CheckMate clinical trials). 1,746 slides were retrospectively analyzed, the largest investigation of digital pathology algorithms on clinical trial datasets performed to date. AI-powered quantification of PD-L1 expression on tumor cells identified more PD-L1–positive samples compared with manual scoring at cutoffs of ≥1% and ≥5% in most tumor types. Additionally, similar improvements in response and survival were observed in patients identified as PD-L1–positive compared with PD-L1–negative using both AI-powered and manual methods, while improved associations with survival were observed in patients with certain tumor types identified as PD-L1–positive using AI-powered scoring only. Our study demonstrates the potential for implementation of digital pathology-based methods in future clinical practice to identify more patients who would benefit from treatment with immuno-oncology therapy compared with current guidelines using manual assessment.

## Introduction

Immuno-oncology therapies, including immune checkpoint inhibitors targeting programmed death-1/death ligand 1 (PD-[L]1) and cytotoxic T lymphocyte antigen-4, have improved clinical outcomes across many tumor types^1–3^. Evidence that PD-L1 expression is a biomarker of response to anti–PD-1/PD-L1 inhibitors has fueled the development and approval of PD-L1 immunohistochemistry (IHC) assays as companion or complementary diagnostics^4–9^. However, manual quantitation of PD-L1 expression can be a laborious and time-consuming process, and while studies indicate moderate to high agreement can be achieved between pathologists^10–13^, there are a number of factors that can lead to reduced inter- and intra-observer reproducibility, particularly at lower cutoff values^10,11,13–15^.

Digital pathology and artificial intelligence (AI)–powered approaches can aid pathologists in overcoming the challenges associated with manual scoring^16–18^. While AI-based methods have demonstrated moderate to high correlation with pathologist scoring in urothelial carcinoma (UC), melanoma (MEL), and breast cancer^19–21^, studies directly comparing their performance in large randomized controlled trials using traditional response and survival endpoints are limited^22^.

In this study, we developed unique AI-powered algorithms to retrospectively evaluate PD-L1 expression on tumor cells (TCs) across multiple tumor types, including samples from patients with non-small cell lung cancer (NSCLC), squamous cell carcinoma of the head and neck (SCCHN), MEL, and UC. The performance of AI-powered analysis was then compared with manual scoring of PD-L1 expression that was prospectively generated as part of phase II and III clinical trials across two different PD-L1 expression cutoffs in patients treated with nivolumab ± ipilimumab (NIVO ± IPI).

## Methods

### Study designs and patients

#### Clinical validation of AI-powered scoring algorithm

Assessment of PD-L1 expression was performed in samples from patients with UC, NSCLC, MEL, and SCCHN treated with NIVO alone from the registrational phase II (CheckMate 275 [NCT02387996]) and phase III (CheckMate 026 [NCT02041533], 057 [NCT01673867], 238 [NCT02388906], 141 [NCT02105636]) clinical trials or NIVO ± IPI from the phase III CheckMate 067 (NCT01844505) trial. The patient demographics and study designs for these trials have been published previously^23–28^.

### Study procedures

#### Clinical assessments

Patient responses were assessed according to Response Evaluation Criteria in Solid Tumors v1.1 as previously described^23–28^. Responses were categorized as complete response, partial response, stable disease, progressive disease, or response not evaluable. Objective response rate (ORR) was calculated using the percentage of patients who achieved a complete or partial response compared with those who achieved stable or progressive disease or were not evaluable. Survival was assessed using overall survival (OS) for CheckMate 057, 275, 067, and 141, recurrence-free survival for CheckMate 238, or progression-free survival for CheckMate 026. For more information regarding survival endpoints in each clinical trial, refer to the Supplementary Methods, “Clinical assessments” section.

#### Sample preparation and biomarker assessment

Formalin-fixed, paraffin-embedded tissue slides were stained using the Dako PD-L1 IHC 28-8 pharmDx assay (Agilent, Santa Clara, CA, USA) per the manufacturer’s instructions as part of the respective clinical trial^23–28^. PD-L1 TC expression was derived from the percentage of TCs with complete circumferential or partial PD-L1 expression at any level of intensity divided by all TCs. For more information regarding PD-L1 testing in each clinical trial, refer to the Supplementary Methods, “PD-L1 assessment in each clinical trial” section.

### Outcomes

#### Development of PD-L1 AI-powered scoring algorithms

To develop a deep-learning model that can generate an AI-powered PD-L1 expression score, whole slide images (WSIs) of PD-L1–stained slides were generated using the Aperio AT2 image-scanning platform (Leica Biosystems, Vista, CA, USA) at 0.5 microns/pixel resolution (20× objective). These WSIs were used to develop tumor-specific algorithms.

Board-certified pathologists from the PathAI network provided more than 250,000 cell-level annotations on a training set of digital WSIs from a mix of commercial and clinical trial biopsy samples from each tumor type stained for PD-L1 expression by IHC. These included 217 samples from patients with NSCLC, 600 from MEL, 400 from SCCHN, and 293 from patients with UC. Annotations defined PD-L1 expression on individual TCs and immune cells (ICs), including macrophages and lymphocytes. For SCCHN and MEL, deep-learning models were trained to recognize and quantify PD-L1–expressing TCs using these annotations while automatically excluding regions that would interfere with PD-L1 scoring, such as areas of background staining, anthracotic pigment, necrosis, areas of poor image quality, and, in the case of MEL samples, areas of melanin filled macrophages (melanophages). With NSCLC and UC samples, the algorithms were trained to recognize areas of background staining, anthracotic pigment, necrosis, etc., as negative for PD-L1 expression. Annotations for normal tissue, tumor parenchyma, and tumor stromal regions were also provided.

Outputs consisting of quantitative features summarizing slide-level PD-L1 expression on TCs were generated for each sample (AI-powered score). Tumor samples were then classified as PD-L1–positive or PD-L1–negative (as described in the previous section), using cutoffs of 1% and 5%. Quality control was performed by board-certified pathologists on tissue samples evaluated for PD-L1 expression. A sample was deemed evaluable if there were ≥100 viable TCs that were in focus and not obscured by artifact or background staining.

**Fig. 1:**
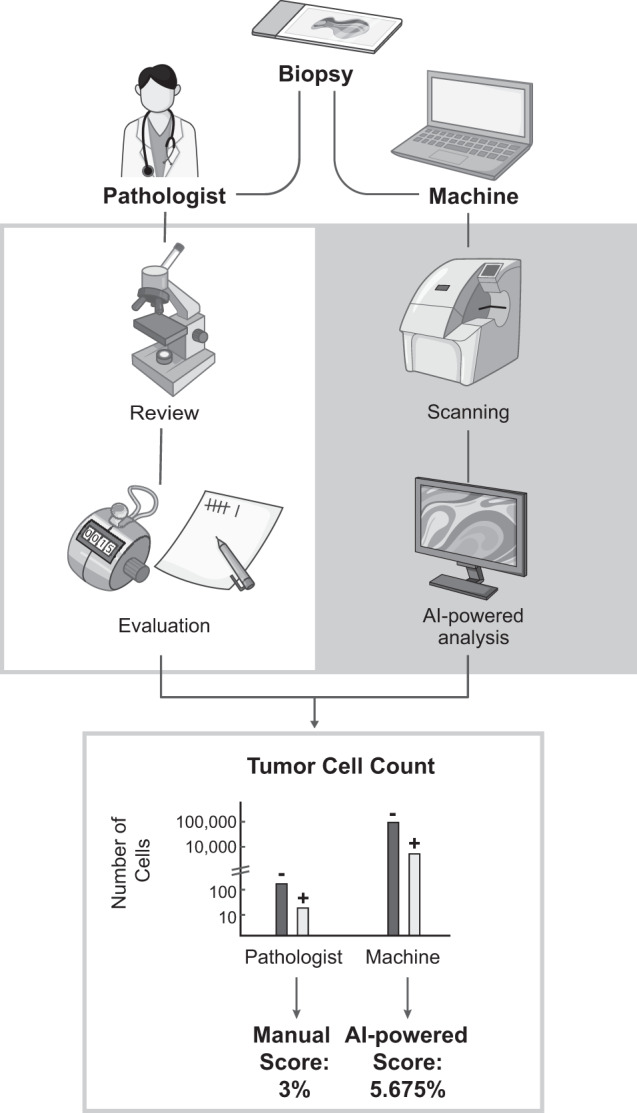
Manual and AI-powered assessment of PD-L1 expression. Manual and AI-powered scores represent % positive PD-L1 TCs. AI artificial intelligence, PD-L1 programmed death ligand 1, TC tumor cell.

To ensure that the overall cell- and tissue-level AI classifications were appropriate, pathologists were asked to review PathAI heatmap overlays of regions of interest and to evaluate whether the algorithm accurately determined TC and IC PD-L1 expression. Each region of interest included tumor, intratumoral stroma, and peritumoral stroma, while areas containing crushed tissue or artifacts were excluded. Pathologists were given thresholds of PD-L1 TC expression to choose from (0–5%, 5–25%, 25–50%, 50%). The AI-powered score was marked as correct if it was within the same threshold as the manual score or as incorrect if it was not. An overview of AI-powered and manual assessment of PD-L1 expression on TCs is provided in Fig. 1.

#### Application of AI-powered algorithm to test set

**Fig. 2:**
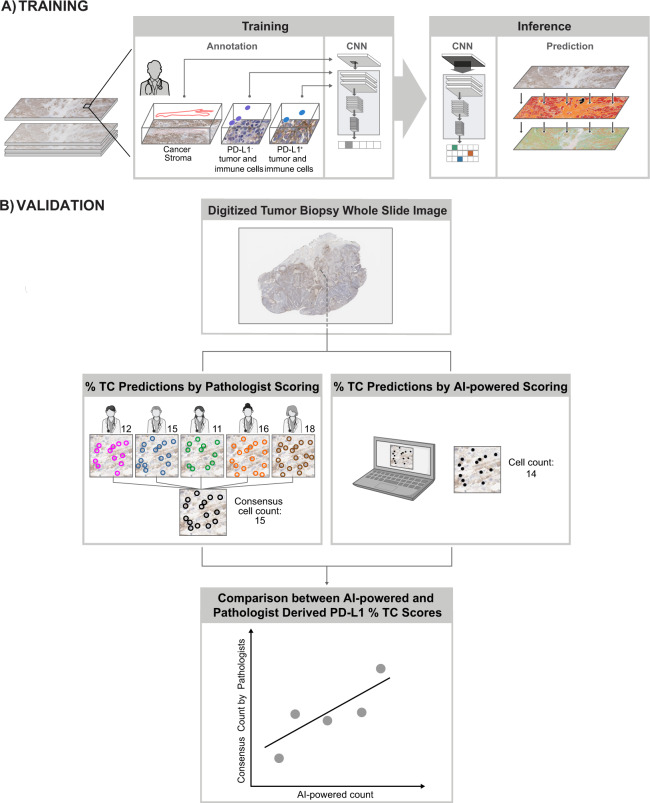
Validation of artificial intelligence–powered scoring. AI artificial intelligence, CNN convolutional neural network, PD-L1 programmed death ligand 1.

Model performance for each tumor type was assessed using an independent set of commercial and clinical trial–procured samples (distinct from the training set) that were stained for PD-L1 expression and digitized using the Aperio AT2 image-scanning platform. High-quality 150 × 150-pixel frames of subregions were defined from WSIs. Exhaustive annotations from five different pathologists from LabCorp (Burlington, NC, USA) were used to classify cell types and identify the absolute number of PD-L1–positive TCs in each frame. Additional details on samples used for training of the AI algorithm can be found in Supplementary Table 1. The median number of PD-L1–positive TCs was used to generate a consensus score. Agreement between the pathologist consensus score and the model-generated PD-L1 score was calculated using Pearson’s correlation coefficients. An overview of this frame-based validation method is provided in Fig. 2.

#### Prevalence of patients with PD-L1–positive tumor samples

Prevalence of PD-L1–positive patients was assessed using both manual and AI-powered scoring at cutoffs of ≥1% and ≥5%. For CheckMate 026, only patients with a PD-L1 expression level of ≥1% underwent randomization and were stratified according to a PD-L1 expression level of < or ≥5%^25^.

### Assessment of AI-powered scoring algorithm across multiple scanners

The AI-powered algorithm, trained and validated as described in the previous “Development of PD-L1 AI-powered scoring algorithms” and “Application of AI-powered scoring algorithm to test set” sections, was used to assess PD-L1 expression on TCs using 20 WSIs from six distinct IHC stained slides. Slides were scanned by two separate Aperio AT2 scanners across 5 days, two times per day (morning [AM] and afternoon [PM]). Five slides represented tumors with a PD-L1 expression ranging from ≥1% to 90% and one slide with a PD-L1 expression level of <1% as a negative control. Tumor samples were obtained from patients with UC as part of the CheckMate 275 clinical trial.

### Statistical analysis

#### Inter-scanner and inter- and intra-day precision

Average and standard deviation (SD) statistics were computed for PD-L1 expression within each group of images pertaining to the same slide scanned at distinct times with different scanners. Analysis of variance tests were performed to determine the significance between the difference in % TC across days, times, and scanners. Coefficients of variation for % TC across all slides were estimated as the SD divided by the mean, multiplied by 100 ([SD/mean]*100).

#### Clinical outcomes

Association of PD-L1 expression on TCs with clinical efficacy was assessed using cutoffs of ≥1% and ≥5%, as evaluated by AI-powered and manual scoring. Kendall’s tau coefficient was used to evaluate the correlation between AI-powered and manual scores within each trial. Odds ratios (ORs) were calculated using logistic regression to examine associations with objective response. Objective response predictions by AI-powered and manual scoring across all trials were assessed by plotting summary receiver operating characteristic curves and calculating the area under the curve (AUC) using metaROC from the R-package with a fully non-parametric estimation with random effect^29^. Hazard ratios were estimated using Cox proportional hazards models to examine associations with progression-free survival, recurrence-free survival, or OS. Kaplan–Meier curves were used to illustrate comparisons of survival in samples identified as positive using both AI-powered and manual scoring, additional samples only identified as positive by AI-powered scoring, additional samples only identified as positive by manual scoring, and samples identified as negative using both AI-powered and manual scoring.

## Results

### Validation of AI-powered scoring algorithm

Validation of the AI-powered scoring algorithm was conducted using a frame-based comparison of AI-powered scoring and pathologist-derived scoring of PD-L1 expression on TCs on WSIs. Using a combination of commercial and clinical tumor samples from patients with NSCLC, SCCHN, MEL, and UC (Supplementary Table 1), AI-based assessment was highly correlated with the median score from manual assessment of PD-L1–expressing TCs by 5 pathologists (*r* ranging from 0.73 to 0.85) and variability fell within the range of pathologists’ scores (Supplementary Fig. 1). We then compared the performance of the AI-based algorithm with manual scoring to evaluate prevalence of PD-L1–positive patients from multiple clinical trials.

### Prevalence of PD-L1 expression by AI-powered and manual scoring

**Fig. 3:**
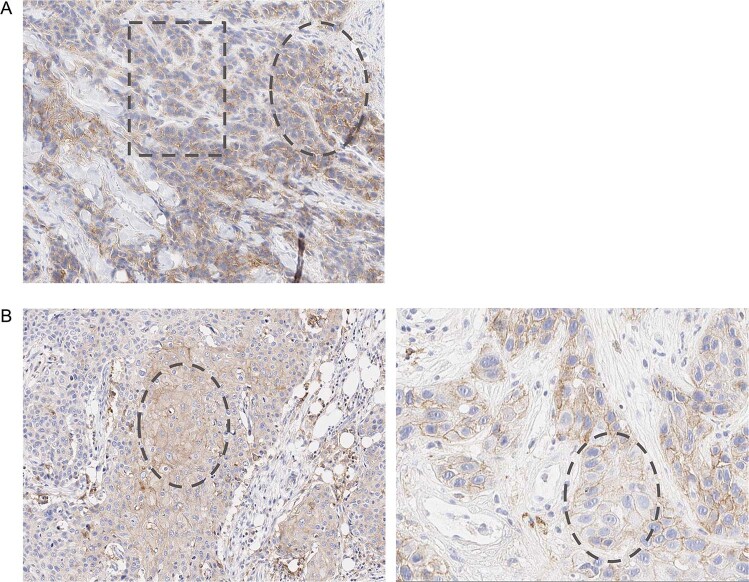
Examples of PD-L1 staining in tissue samples from patients with SCCHN. **A** Illustrates low PD-L1 membrane staining in basaloid SCCHN (oval outline) with preparation artifact as outlined by the square (decreased sharpness of nuclei borders and reduced clarity of inner chromatin structure; 3,3′-Diaminobenzidine reactivity [purple staining] is blurred and indistinct from artifacts), shown at 19× magnification. **B** Illustrates cytoplasmic positivity (blush) (oval outline in left-hand image) at 14× magnification coincident with weak (1+) membrane PD-L1–positive staining (oval outline in right-hand image) at 30× magnification. PD-L1 programmed death ligand 1.

The algorithm tended to identify a higher prevalence of PD-L1–positive patients as compared with manual scoring. This trend was observed across the majority of tumor types (Table 1) and was consistent across both the 1% and 5% cutoffs. In patients with NSCLC (CheckMate 057), UC (CheckMate 275), and MEL (CheckMate 067 and 238), the prevalence of PD-L1–positive patients increased in the range of 5% to 39% and from 6% to 25% with AI-powered scoring compared with manual scoring at PD-L1 expression cutoffs of ≥1% and ≥5%, respectively. In patients with SCCHN (CheckMate 141), a lower prevalence of PD-L1–positive patients was seen with AI-powered scoring (42.5% and 28.8%) compared with manual scoring (54.9% and 34.0%) at cutoffs of ≥1% and ≥5%, respectively, though the difference was not significant (Table 1). This could be due to a number of factors, such as presence of crush artifact or low PD-L1 membrane staining with cytoplasmic positivity (blush) (see Fig. 3 and Discussion).

**Table 1:** Prevalence of PD-L1 expression by artificial intelligence-powered and manual scoring.

	Evaluable samples for AI-powered assessment—no.	Prevalence—no. (%)				*P* value
		AI-powered^a^		Manual^a^		
**1% cutoff**						
		<1%	≥1%	<1%	≥1%	
CheckMate 057 (NSCLC)	194	19 (9.8)	175 (90.2)	95 (49.0)	99 (51.0)	<0.01
CheckMate 275 (UC)	241	75 (30.1)	166 (68.9)	128 (53.1)	113 (46.8)	<0.01
CheckMate 067 (MEL; NIVO)	265	91 (34.3)	174 (65.7)	104 (39.3)	161 (60.8)	0.28
CheckMate 067 (MEL; NIVO + IPI)	262	101 (38.6)	161 (61.5)	117 (44.7)	145 (55.3)	0.18
CheckMate 238 (MEL)	377	70 (18.6)	307 (81.4)	118 (31.3)	259 (68.7)	<0.01
CheckMate 141 (SCCHN)	153	88 (57.5)	65 (42.5)	69 (45.1)	84 (54.9)	0.04
**5% cutoff**						
		<5%	≥5%	<5%	≥5%	
CheckMate 026 (NSCLC)	254	21 (8.3)	233 (91.7)	58 (22.8)	196 (77.2)	<0.01
CheckMate 057 (NSCLC)	194	84 (43.3)	110 (56.7)	117 (60.3)	77 (39.7)	<0.01
CheckMate 275 (UC)	241	151 (62.7)	90 (37.3)	167 (69.3)	74 (30.7)	0.15
CheckMate 067 (MEL; NIVO)	265	162 (61.1)	103 (38.9)	189 (71.3)	76 (28.7)	0.02
CheckMate 067 (MEL; NIVO + IPI)	262	170 (64.9)	92 (35.1)	197 (75.2)	65 (24.8)	0.01
CheckMate 238 (MEL)	377	143 (37.9)	234 (62.1)	238 (63.1)	139 (36.9)	<0.01
CheckMate 141 (SCCHN)	153	109 (71.2)	44 (28.8)	101 (66.0)	52 (34.0)	0.39

Given the observed trend for higher prevalence with the AI-powered scoring algorithm, we assessed whether this affected prediction of treatment response.

### Comparison of AI-powered and manual scoring as predictors of response

**Fig. 4:**
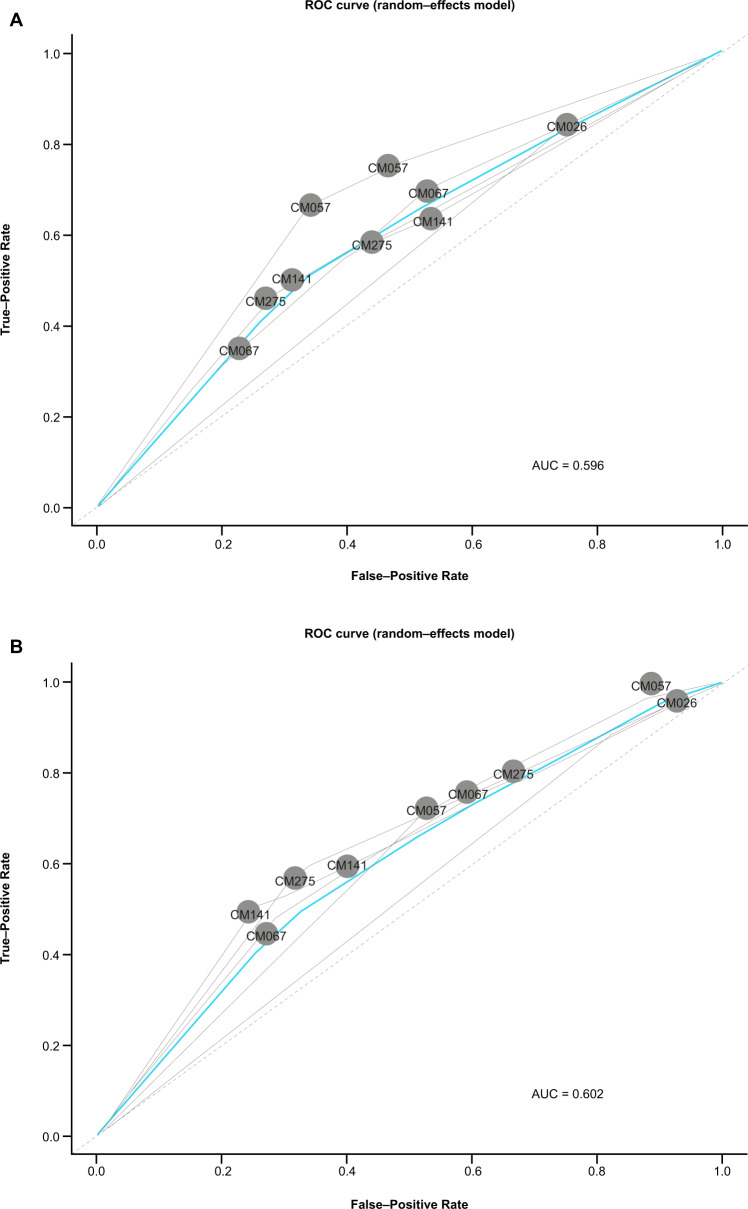
Comparison of artifical intelligence–powered and manual scoring as a predictor of ORR across trials. **A** Artificial intelligence-powered scoring. **B** Manual scoring. Associations of each trial population at the 1% and 5% cutoffs to ORR were plotted in the gray solid lines. Dotted line is the null reference representing a classifier predicting association of PD-L1 expression with random chance. Fitted sROC and 95% confidence intervals are drawn in blue. For CheckMate 026, only patients with a PD-L1 expression level ≥1% underwent randomization and were stratified according to a PD-L1 expression level of < or ≥5%. No response data are available for the adjuvant CheckMate 238 study, which was therefore excluded from this analysis. AUC area under curve, ORR objective response rate, PD-L1 programmed death ligand 1, sROC summary receiver operating characteristic.

The combined sensitivity and specificity of AI-powered and manual scoring for predicting ORR was assessed for all trials and both PD-L1 expression cutoffs used in this study. AUC values derived from summary receiver operating characteristic curves were similar for AI-powered (AUC = 0.602) (Fig. 4A) and manual scoring (AUC = 0.596) (Fig. 4B), suggesting that the performance of each scoring method was similar in predicting ORR.

### Association of PD-L1 expression with ORR

**Fig. 5:**
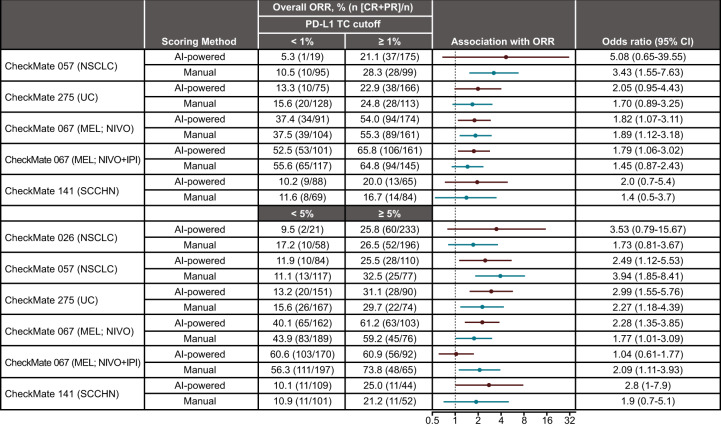
Association of objective response with PD-L1 expression as assessed by artificial intelligence-powered and manual scoring methods. For CheckMate 026, only patients with a PD-L1 expression level ≥1% underwent randomization and were stratified according to a PD-L1 expression level of < or ≥5%. No response data are available for the adjuvant CheckMate 238 study, which was therefore excluded from this analysis. CI confidence interval, CR complete response, IPI ipilimumab, MEL melanoma, NIVO nivolumab, NSCLC non-small cell lung cancer, ORR objective response rate, PD-L1 programmed death ligand 1, PR partial response, SCCHN squamous cell carcinoma of the head and neck, TC tumor cell, UC urothelial carcinoma.

To assess the potential impact, including increased prevalence, of AI-powered scoring on ORR, we reanalyzed PD-L1 expression in each study at the predefined cutoffs of ≥1% and ≥5% using AI-powered assessment and directly compared the value with that obtained using manual scoring as part of the original trial. In general, the majority of OR point estimates suggested a slight increase in the association between ORR and patients identified as PD-L1–positive using AI-powered scoring compared with manual scoring in four out of five studies (NSCLC [CheckMate 057], UC [CheckMate 275], MEL [CheckMate 067 NIVO + IPI arm], and SCCHN [CheckMate 141]) at the 1% cutoff and in four out of six studies (NSCLC [CheckMate 026], UC [CheckMate 275], MEL [CheckMate 067 NIVO arm], and SCCHN [CheckMate 141]) at the 5% cutoff. However, there was no significant statistical impact on any of the OR confidence bounds (Fig. 5).

In all but three studies, ORRs in patients identified as PD-L1–positive were similar, regardless of the AI-powered or manual method used, suggesting that AI-powered assessment can correctly identify PD-L1–positive patients who would respond to and thereby benefit from immuno-oncology therapy. The exceptions included CheckMate 057 (NSCLC), where patients identified as PD-L1–positive at cutoffs of ≥1% and ≥5% using AI-powered scoring were associated with a lower ORR (21.1% and 25.5%) compared with manual scoring (28.3% and 32.5%) (Fig. 5). Likewise, in the NIVO + IPI arm of CheckMate 067 (MEL), ORR increased when assessed by manual scoring (73.8%) compared with AI-powered scoring (60.9%) at a cutoff of ≥5%. Conversely, in CheckMate 141 (SCCHN), there was a slight increase in ORR in patients identified as PD-L1–positive using AI-powered scoring (20.0% and 25.0%) compared with manual scoring (16.7% and 21.2%) at cutoffs of ≥1% and ≥5% (Fig. 5). We then determined the impact of AI-powered scoring on survival outcomes as determined in each clinical trial.

### Association of PD-L1 expression with survival

**Fig. 6:**
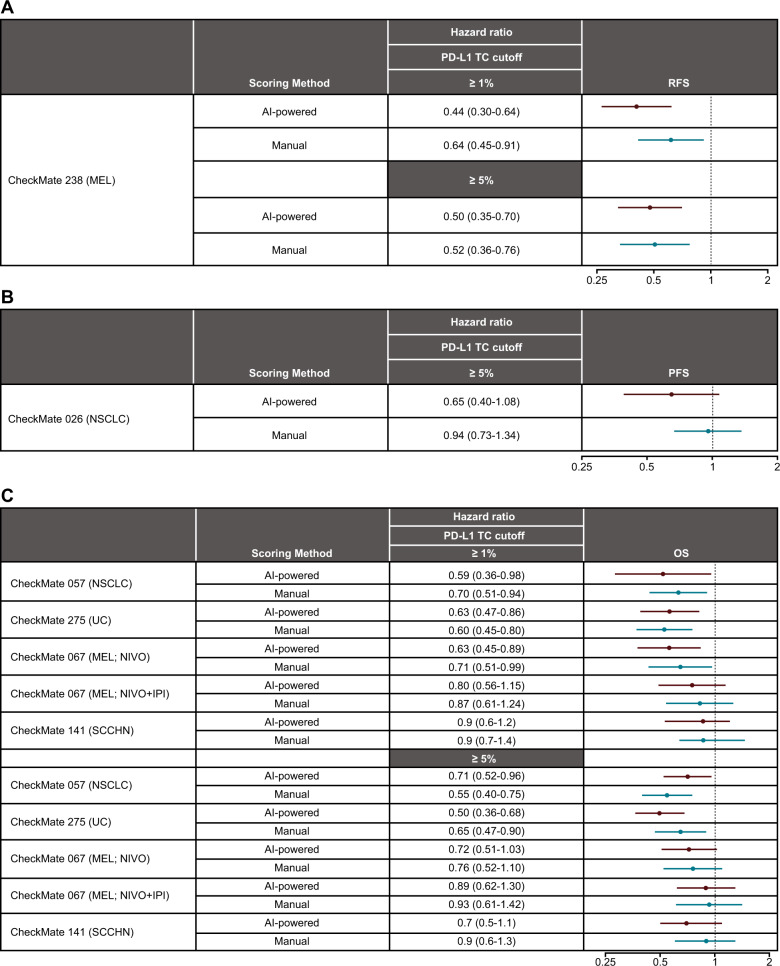
Association of survival with PD-L1 expression by artificial intelligence-powered and manual scoring. **A** RFS. **B** OS. **C** PFS. For CheckMate 026, only patients with a PD-L1 expression level ≥1% underwent randomization and were stratified according to a PD-L1 expression level of < or ≥5%. Therefore, results for the 1% cutoff are not shown. HR hazard ratio, IPI ipilimumab, MEL melanoma, NIVO nivolumab, NSCLC non-small cell lung cancer, ORR objective response rate, OS overall survival, PD-L1 programmed death ligand 1, PFS progression-free survival, RFS recurrence-free survival, SCCHN squamous cell carcinoma of the head and neck, TC tumor cell, UC urothelial carcinoma.

PD-L1 expression on TCs at cutoffs of ≥1% and ≥5% assessed using AI-powered or manual scoring was significantly associated with recurrence-free survival in NIVO-treated patients with MEL (CheckMate 238) (Fig. 6A). In patients with NSCLC (CheckMate 026) identified as PD-L1–positive by either AI-powered or manual scoring, progression-free survival was prolonged at a cutoff of ≥5% in patients treated with NIVO (Fig. 6B). Additionally, PD-L1 expression assessed by either method was significantly associated with OS in patients with NSCLC (CheckMate 057) and UC (CheckMate 275) at both cutoffs (Fig. 6C). In patients with MEL (CheckMate 067) treated with NIVO, both methods were significantly associated with OS at a cutoff of ≥1%, but not at the ≥5% cutoff. In the same trial, no association with OS was seen at either cutoff using both AI and manual PD-L1 methods in patients treated with NIVO + IPI (Fig. 6C). In patients with SCCHN, OS benefit was similar for PD-L1–positive patients identified by AI-powered and manual scoring (Fig. 6C).

Across all tumor types and cutoffs, patients identified as PD-L1–positive by both manual and AI-powered scoring demonstrated improved associations with survival compared with patients identified as PD-L1–negative by both manual and AI-powered assessment. Additionally, patients identified as PD-L1–positive by AI-powered scoring alone demonstrated improved associations with survival compared with those identified as PD-L1–negative by both methods in some tumor types and clinical trials across different cutoffs (Supplementary Fig. 2).

### Analytical precision of AI-powered scoring algorithm

#### Inter- and intra-reproducibility of the AI-powered scoring algorithm

**Fig. 7:**
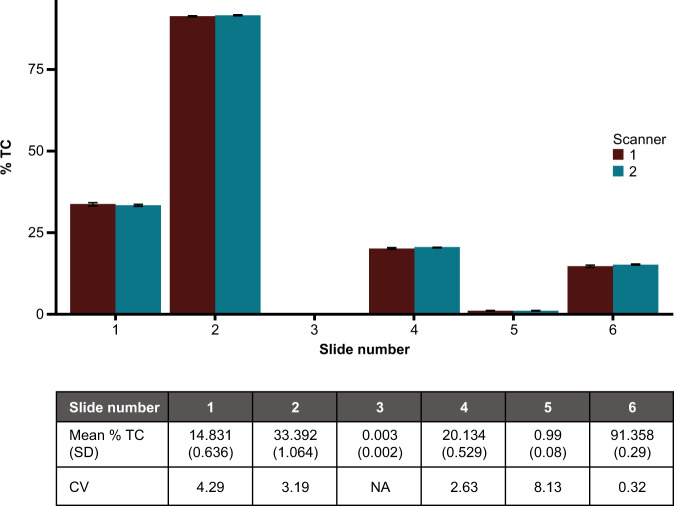
Variation across mean PD-L1 % TC values across 6 slides from patients with UC scanned with two different scanners. Top graph: Bars show mean % TC values across 10 scans with each scanner. Error bars represent SD. Bottom table: Means, SDs, and CVs for % TC values for each slide pooled from 20 scans across both scanners. CV coefficient of variation, NA not applicable, PD-L1 programmed death ligand 1, SD standard deviation, TC tumor cell.

To assess whether our algorithm can produce consistent results when WSIs are obtained from multiple slides scanned using different scanners, we evaluated the inter-scanner precision. No significant variation in % TC values obtained from each slide scanned with either scanner 1 or scanner 2 was observed (Fig. 7). Additionally, mean % TC values did not significantly differ between different days in which slides were scanned (*p* > 0.05) or at different times on the same day (*p* > 0.05).

## Discussion

Recent approvals of immune checkpoint inhibitors with companion PD-L1 IHC assays in various tumor types demonstrate the increasing utility and widespread clinical use of PD-L1 testing to determine patients who may benefit from these therapies. However, classification and stratification of patients based on manual IHC methods may not always be reproducible, as a number of factors can create challenges for pathologists when scoring PD-L1 on TCs, such as heterogenous PD-L1 expression within the tumor microenvironment and variable staining patterns in different cellular compartments (e.g. membrane vs. cytoplasmic staining), potentially leading to substantial inter-observer variability^6,15,17,30,31^. As demonstrated by our frame-based validation method, the results of AI-powered scoring were shown to be comparable to manual assessment of PD-L1 expression on TCs and fell within the variability limits of human error observed across pathologists. However, the reproducibility of AI-powered scoring can reduce inter-observer variability and subjectivity while potentially increasing sensitivity and specificity when scoring and interpreting stains^16^. In studies using manual scoring as the reference standard, an AI-powered approach has been shown to increase inter-observer reproducibility and accuracy of biomarker scoring in breast cancer, NSCLC, and MEL samples, leading to better identification of patients who may benefit from ICI therapy^16,17,21,32–34^.

AI-powered scoring can also be applied to algorithms that include ICs, such as combined positive score^35^. In these algorithms, PD-L1 IC expression can be difficult to reliably assess visually, and thus pathologist concordance tends to be lower^13,14,36,37^. AI-powered scoring methods may thereby offer more precise and consistent results when defining PD-L1 expression on both TCs and ICs across multiple tumor types and cutoffs. However, despite these advantages, there is a reluctance to utilize digital pathology approaches in biomarker scoring and drug development, due to a lack of large-scale clinical validation studies in the oncology setting.

Of relevance to this current study, associations of manually scored TC PD-L1 expression with clinical benefit of NIVO ± IPI have been studied across multiple tumor types and PD-L1 expression cutoffs with varying results^23–27,38^. Given the development of AI-based IHC quantitation methods and their potential for scalability and use in routine clinical practice, we sought to evaluate the performance of an AI-based algorithm to quantify PD-L1 expression using samples from several pivotal trials evaluating NIVO ± IPI across multiple tumor types. In one of the largest sample sizes to date (*n* = 1746), we assessed both AI-based and manual quantification of PD-L1 expression on TCs and compared their associations with response and survival.

We found that more patients with PD-L1 expression at cutoffs of ≥1% and ≥5% were identified by AI-powered scoring compared with manual scoring in patients with NSCLC, UC, and MEL. This increase in measured prevalence of positive patients using AI-based method is likely a result of multiple factors. The algorithm exhaustively analyzes and classifies every cell on the tissue image, thereby providing a highly precise measure of the true PD-L1 positivity on TCs. Although the algorithm is extensively evaluated for accuracy in cell classification, some level of misclassification is expected. In general, the observed discordances between manual and AI-powered scoring were associated with multiple factors. In certain scenarios, the model correctly identifies TCs but does not classify them as PD-L1–positive. These misclassifications could be due to factors such as the presence of clustered, membranous PD-L1–positive TCs overlapping with PD-L1–negative TCs or misclassification of PD-L1–positive ICs as PD-L1–positive TCs. In our frames-based validation analysis shown in Supplementary Fig. 1, we have observed certain discordant frames with examples of both the model and the pathologists overestimating the number of PD-L1–positive TCs. Based on this analysis, such errors were relatively low, and any sample with a large number of misclassifications was flagged during the quality control process. Conversely, a higher prevalence of PD-L1–positive samples was identified by manual scoring compared with AI-powered scoring at both cutoffs in patients with SCCHN. Interpreting PD-L1 expression requires reproducibility across the spectrum of SCCHN differentiation. Manual assessment of PD-L1 expression in basaloid or poorly differentiated SCCHN tumors can be challenging, due to issues such as crush artifacts from tissue handling; such cases may be accurately identified as PD-L1–positive by manual scoring but misclassified as PD-L1–negative by the algorithm. Additionally, non-specific cytoplasmic blush staining coincident with weak membrane PD-L1–positive staining may lead to under-detection of membrane staining by the stringent AI model developed for SCCHN (examples of these can be found in Fig. 2). Another challenge pertaining to assessment of PD-L1 expression in moderate to well-differentiated SCCHN is the presence of keratinized degenerate and anucleate cells, which may be identified as PD-L1–positive by manual scoring but as PD-L1–negative by the algorithm. The model was intentionally trained to reduce false positive detection due to these factors, with a consequent decrease in the detection of low membrane staining of PD-L1, especially in basaloid variant tumors. However, the algorithm sufficiently identified the majority of the responders in CheckMate 141, consistent with manual scoring, as demonstrated by the similar ORR in patients identified as PD-L1–positive using AI-powered scoring as compared with manual scoring. This demonstrates the need for the development of algorithms optimized to account for morphological features unique to each tumor type.

We then assessed clinical endpoints to determine if the increase in prevalence of patients identified as PD-L1–positive using AI-powered scoring was associated with clinical benefit. In evaluated patients with NSCLC, UC, and MEL, treatment response and survival were similar in patients identified as PD-L1–positive using either AI-powered or manual scoring, suggesting that the additional PD-L1–positive patients identified using AI-powered scoring had a similar treatment response and survival to those identified as PD-L1–positive by both methods. AI-powered scoring of PD-L1 expression may therefore detect patients with PD-L1–positive tumors that express low levels of PD-L1, which may go undetected by manual scoring methods.

Finally, we conducted a separate analysis using our previously trained and validated algorithm to assess the reproducibility of AI-powered scoring of PD-L1 expression across different scanners, days, and times of day. No significant variations in identification of PD-L1–positive TCs based on day, time of day, or scanner were identified. These results demonstrate the ability of the AI algorithm to overcome analytical factors that may occur during a typical workflow to produce consistent and accurate results.

To our knowledge, this is the first study utilizing a large sample size across various tumor types to develop and compare the ability of AI-based scoring and manual assessment to identify PD-L1 expression on TCs and its association with clinical efficacy in a large cohort of patients from multiple trials. Previous studies on single tumor types with a small number of patients have also sought to compare digital and manual assessment of PD-L1 expression. Koelzer et al. sought to create a standardized digital protocol for the assessment of PD-L1 staining in MEL (*n* = 69) and to compare the output data and reproducibility to conventional assessment by expert pathologists. Consistent with our results, high correlation was observed between digital and manual assessment in MEL samples. Additionally, the image analysis protocol had high inter-reader reproducibility and reduced variability compared with manual assessment of PD-L1 expression^33^. Another study compared the results of PD-L1 expression using combined positive score in samples from a small phase II trial in patients with gastric cancer (*n* = 39), as measured by digital image analysis and pathologist interpretation, and its ability to predict response to pembrolizumab. Similar to our findings, both methods were predictive for response to pembrolizumab in patients with gastric cancer. However, there are some important differences in this study compared with our study, including use of a small set of samples from one clinical trial and the inability of the image analysis tool to distinguish between PD-L1–positive TCs and ICs, that limit the ability to determine the respective role of each cell type in predicting response^39^.

This investigation has limitations due to the retrospective nature of our treatment response and survival analyses. Additionally, since we sought to compare AI-powered scoring with manual scoring carried out as part of the original trials, the majority of which did not assess PD-L1 positivity in immune compartments, we limited our analysis to evaluating PD-L1 expression on TCs only. Therefore, our results cannot be extrapolated to other scoring methods or assays. However, our scoring algorithm has the potential to be used to determine staining in additional cell types^19,40^ and warrants further study to include additional scoring methods that incorporate assessment of ICs, such as combined positive score, and application in additional tumor types.

Our study demonstrates that AI-powered quantification of PD-L1 expression on TCs identified more PD-L1–positive samples compared with manual scoring across several tumor types explored in this study, while demonstrating consistent associations with response and survival across multiple clinical trial datasets. Compared with manual scoring, our AI algorithm has the potential to identify more patients who may benefit from immuno-oncology therapy. The findings of our study could serve as a framework for incorporation of AI-powered scoring as a precise, reproducible, scalable, and exhaustive approach to quantifying PD-L1 expression on TCs in routine practice, leading the way for application in future prospective large-scale clinical trials.

## Supplementary information

Supplementary Information

## Data Availability

Any additional data not included in the manuscript or supplementary files that support the findings of this study are available from the corresponding author VB.

## References

[1] Herbst RS, Baas P, Kim D-W, Felip E, Pérez-Gracia JL, Han J-Y, et al. Pembrolizumab versus docetaxel for previously treated, PD-L1-positive, advanced non-small-cell lung cancer (KEYNOTE-010): a randomised controlled trial. *Lancet*. **387**, 1540–1550(2016) 10.1016/S0140-6736(15)01281-7 26712084

[2] Balar AV, Galsky MD, Rosenberg JE, Powles T, Petrylak DP, Bellmunt J, et al. Atezolizumab as first-line treatment in cisplatin-ineligible patients with locally advanced and metastatic urothelial carcinoma: a single-arm, multicentre, phase 2 trial. *Lancet*. **389**, 67–76(2017) 10.1016/S0140-6736(16)32455-2 PMC5568632 27939400

[3] Larkin J, Chiarion-Sileni V, Gonzalez R, Grob J-J, Rutkowski P, Lao CD, et al. Five-year survival with combined nivolumab and ipilimumab in advanced melanoma. *N Engl J Med*. **381**, 1535–1546(2019) 10.1056/NEJMoa1910836 31562797

[4] Schmid P, Adams S, Rugo HS, Schneeweiss A, Barrios CH, Iwata H, et al. Atezolizumab and nab-paclitaxel in advanced triple-negative breast cancer. *N Engl J Med*. **379**, 2108–2121(2018) 10.1056/NEJMoa1809615 30345906

[5] Mok TSK, Wu Y-L, Kudaba I, Kowalski DM, Chul Cho B, Turna HZ, et al. Pembrolizumab versus chemotherapy for previously untreated, PD-L1-expressing, locally advanced or metastatic non-small-cell lung cancer (KEYNOTE-042): a randomised, open-label, controlled, phase 3 trial. *Lancet*. **393**, 1819–1830(2019) 10.1016/S0140-6736(18)32409-7 30955977

[6] Büttner R, Gosney JR, Skov BG, Adam J, Motoi N, Bloom KJ, et al. Programmed death-ligand 1 immunohistochemistry testing: A review of analytical assays and clinical implementation in non-small-cell lung cancer. *J Clin Oncol*. **35**, 3867-3876(2017) 10.1200/JCO.2017.74.7642 29053400

[7] Hellmann MD, Paz-Ares L, Bernabe Caro R, Zurawski B, Kim S-W, Carcereny Costa E, et al. Nivolumab plus ipilimumab in advanced non–small-cell lung cancer. *N Engl J Med*. **381**, 2020–2031(2019) 10.1056/NEJMoa1910231 31562796

[8] Phillips T, Simmons P, Inzunza HD, Cogswell J, Novotny Jr J, Taylor C, et al. Development of an automated PD-L1 immunohistochemistry (IHC) assay for non-small cell lung cancer. *Appl Immunohistochem Mol Morphol*. **23**, 541-549(2015) 10.1097/PAI.0000000000000256 PMC4561627 26317305

[9] Cogswell J, Inzunza HD, Wu Q, Feder JN, Mintier G, Novotny J, et al. An analytical comparison of Dako 28-8 pharmDx assay and an E1L3N laboratory-developed test in the immunohistochemical detection of programmed death-ligand 1. *Mol Diagn Ther*. **21**, 85-93(2017) 10.1007/s40291-016-0237-9 PMC5250639 27667773

[10] Adam J, Hofman V, Mansuet-Lupo A, Rouquette I, Vignaud J, Badoual C, et al. P2.09-17 real-world concordance across pathologists for PD-L1 scoring in non-small cell lung cancer: Results from a large nationwide initiative. *J Thorac Oncol*. **14**, S775 (2019)

[11] Chang S, Park HK, Choi Y-L, Jang SJ, Cardiopulmonary Pathology Study Group of the Korean Society of Pathologists. Interobserver reproducibility of PD-L1 biomarker in non-small cell lung cancer: a multi-institutional study by 27 pathologists. *J Pathol Transl Med*. **53**, 347-353(2019) 10.4132/jptm.2019.09.29 PMC6877442 31656061

[12] Cooper WA, Russell PA, Cherian M, Duhig EE, Godbolt D, Jessup PJ, et al. Intra- and interobserver reproducibility assessment of PD-L1 biomarker in non–small cell lung cancer. *Clin Cancer Res*. **23**, 4569-4577(2017) 10.1158/1078-0432.CCR-17-0151 28420726

[13] Tsao MS, Kerr KM, Kockx M, Beasley MB, Borczuk AC, Botling J, et al. PD-L1 immunohistochemistry comparability study in real-life clinical samples: results of Blueprint phase 2 project. *J Thorac Oncol*. **13**, 1302–1311(2018) 10.1016/j.jtho.2018.05.013 PMC8386299 29800747

[14] Prince EA, Sanzari JK, Pandya D, Huron D, Edwards R. Analytical concordance of PD-L1 assays utilizing antibodies from FDA-approved diagnostics in advanced cancers: A systematic literature review. *JCO Precis Oncol*. **5**, 953–973(2021) 10.1200/PO.20.00412 PMC8202559 34136742

[15] Brunnström H, Johansson A, Westbom-Fremer S, Backman M, Djureinovic D, Patthey A, et al. PD-L1 immunohistochemistry in clinical diagnostics of lung cancer: inter-pathologist variability is higher than assay variability. *Mod Pathol*. **30**, 1411-1421(2017) 10.1038/modpathol.2017.59 28664936

[16] Bera K, Schalper KA, Rimm DL, Velcheti V, Madabhushi A. Artificial intelligence in digital pathology - new tools for diagnosis and precision oncology. *Nat Rev Clin Oncol*. **16**, 703–715(2019) 10.1038/s41571-019-0252-y PMC6880861 31399699

[17] Koelzer VH, Sirinukunwattana K, Rittscher J, Mertz KD. Precision immunoprofiling by image analysis and artificial intelligence. *Virchows Arch*. **474**, 511-522(2019) 10.1007/s00428-018-2485-z PMC6447694 30470933

[18] Kapil A, Meier A, Zuraw A, Steele KE, Rebelatto MC, Schmidt G, et al. Deep semi supervised generative learning for automated tumor proportion scoring on NSCLC tissue needle biopsies. *Sci Rep*. **8**, 17343(2018) 10.1038/s41598-018-35501-5 PMC6255873 30478349

[19] Beck A, Glass B, Elliott H, Kerner JK, Khosla A, Lahiri A, et al. P730 An empirical framework for validating artificial intelligence-derived PD-L1 positivity predictions applied to urothelial carcinoma. *J Immunother Cancer*. **7**(**suppl 1**), 283(2019)

[20] Duan C, Montalto M, Lee G, Pandya D, Cohen D, Chang H, et al. Abstract 2017: Association of digital and manual quantification of tumor PD-L1 expression with outcomes in nivolumab-treated patients. *Cancer Res*. **80**, 2017-2017 (2020)

[21] Barnes M, Srinivas C, Bai I, Frederick J, Liu W, Sarkar A, et al. Whole tumor section quantitative image analysis maximizes between-pathologists’ reproducibility for clinical immunohistochemistry-based biomarkers. *Lab Invest*. **97**, 1508-1515(2017) 10.1038/labinvest.2017.82 28805805

[22] Althammer S, Tan TH, Spitzmüller A, Rognoni L, Wiestler T, Herz T, et al. Automated image analysis of NSCLC biopsies to predict response to anti-PD-L1 therapy. *J Immunother Cancer*. **7**, 121(2019) 10.1186/s40425-019-0589-x PMC6501300 31060602

[23] Borghaei H, Paz-Ares L, Horn L, Spigel DR, Steins M, Ready NE, et al. Nivolumab versus docetaxel in advanced nonsquamous non-small-cell lung cancer. *N Engl J Med*. **373**, 1627–1639(2015) 10.1056/NEJMoa1507643 PMC5705936 26412456

[24] Weber J, Mandalá M, Del Vecchio M, Gogas HJ, Arance AM, Cowey CL, et al. Adjuvant nivolumab versus ipilimumab in resected stage III or IV melanoma. *N Engl J Med*. **377**, 1824-1835(2017) 10.1056/NEJMoa1709030 28891423

[25] Carbone DP, Reck M, Paz-Ares L, Creelan B, Horn L, Steins M, et al. First-line nivolumab in stage IV or recurrent non-small-cell lung cancer. *N Engl J Med*. **376**, 2415–2426(2017) 10.1056/NEJMoa1613493 PMC6487310 28636851

[26] Ferris RL, Blumenschein Jr G, Fayette J, Guigay J, Colevas AD, Licitra L, et al. Nivolumab for recurrent squamous-cell carcinoma of the head and neck. *N Engl J Med*. **375**, 1856–1867(2016) 10.1056/NEJMoa1602252 PMC5564292 27718784

[27] Sharma P, Retz M, Siefker-Radtke A, Baron A, Necchi A, Bedke J, et al. Nivolumab in metastatic urothelial carcinoma after platinum therapy (CheckMate 275): a multicentre, single-arm, phase 2 trial. *Lancet Oncol*. **18**, 312–322(2017) 10.1016/S1470-2045(17)30065-7 28131785

[28] Larkin J, Chiarion-Sileni V, Gonzalez R, Grob JJ, Cowey CL, Lao CD, et al. Combined nivolumab and ipilimumab or monotherapy in previously untreated melanoma. *N Engl J Med*. **373**, 23–34(2015) 10.1056/NEJMoa1504030 PMC5698905 26027431

[29] Martinez-Camblor P. Fully non-parametric receiver operating characteristic curve estimation for random-effects meta-analysis. *Stat Methods Med Res*. **26**, 5-20(2017) 10.1177/0962280214537047 24872352

[30] Noguchi T, Ward JP, Gubin MM, Arthur CD, Lee SH, Hundal J, et al. Temporally distinct PD-L1 expression by tumor and host cells contributes to immune escape. *Cancer Immunol Res*. **5**, 106-117(2017) 10.1158/2326-6066.CIR-16-0391 PMC5510474 28073774

[31] Rimm DL, Han G, Taube JM, Yi ES, Bridge JA, Flieder DB, et al. A prospective, multi-institutional, pathologist-based assessment of 4 immunohistochemistry assays for PD-L1 expression in non-small cell lung cancer. *JAMA Oncol*. **3**, 1051–1058(2017) 10.1001/jamaoncol.2017.0013 PMC5650234 28278348

[32] Kearney S, Black J, Aeffner F, Black J, Pratte L, Krueger J. Abstract 4582: Evaluating benefits of PD-L1 image analysis for the clinical setting. *Cancer Res*. **77****(****suppl 13**), 4582 (2017)

[33] Koelzer VH, Gisler A, Hanhart JC, Griss J, Wagner SN, Willi N, et al. Digital image analysis improves precision of PD-L1 scoring in cutaneous melanoma. *Histopathology*. **73**, 397-406(2018) 10.1111/his.13528 29660160

[34] Taylor CR, Jadhav AP, Gholap A, Kamble G, Huang J, Gown A, et al. A multi-institutional study to evaluate automated whole slide scoring of immunohistochemistry for assessment of programmed death-ligand 1 (PD-L1) expression in non-small cell lung cancer. *Appl Immunohistochem Mol Morphol*. **27**, 263-269(2019) 10.1097/PAI.0000000000000737 30640753

[35] Kulangara K, Zhang N, Corigliano E, Guerrero L, Waldroup S, Jaiswal D, et al. Clinical utility of the combined positive score for programmed death ligand-1 expression and the approval of pembrolizumab for treatment of gastric cancer. *Arch Pathol Lab Med*. **143**, 330-337(2019) 10.5858/arpa.2018-0043-OA 30028179

[36] Feng Z, Jensen SM, Messenheimer DJ, Farhad M, Neuberger M, Bifulco CB, et al. Multispectral imaging of T and B cells in murine spleen and tumor. *J Immunol*. **196**, 3943-3950(2016) 10.4049/jimmunol.1502635 PMC4834492 26994219

[37] Reisenbichler ES, Han G, Bellizzi A, Bossuyt V, Brock J, Cole K, et al. Prospective multi-institutional evaluation of pathologist assessment of PD-L1 assays for patient selection in triple negative breast cancer. *Mod Pathol*. **33**, 1746–1752 (2020) 10.1038/s41379-020-0544-x PMC8366569 32300181

[38] Wolchok JD, Chiarion-Sileni V, Gonzalez R, Rutkowski P, Grob J-J, Cowey CL, et al. Overall survival with combined nivolumab and ipilimumab in advanced melanoma. *N Engl J Med*. **377**, 1345–1356(2017) 10.1056/NEJMoa1709684 PMC5706778 28889792

[39] Kim H-N, Jang J, Heo YJ, Kim B, Jung H, Jang Y, et al. PD-L1 expression in gastric cancer determined by digital image analyses: pitfalls and correlation with pathologist interpretation. *Virchows Arch*. **476**, 243-250(2020) 10.1007/s00428-019-02653-2 31463730

[40] Baxi V, Beck A, Pandya D, Lee G, Hedvat C, Khosla A, et al. O65 Artificial intelligence–powered retrospective analysis of PD-L1 expression in nivolumab trials of advanced non-small cell lung cancer. *J Immunother Cancer*. **7** (**suppl 1**), 283 (2019)

