# Supplementary material for: Association of artificial intelligence-powered and manual quantification of programmed death-ligand 1 (PD-L1) expression with outcomes in patients treated with nivolumab ± ipilimumab
Source: Mod Pathol. 2022 Jul 15;35(11):1529–39. doi: 10.1038/s41379-022-01119-2 (PMC9596372; doi:10.1038/s41379-022-01119-2)
Supplement: Supplementary file 1 — Supplementary Information [file 41379_2022_1119_MOESM1_ESM.docx]

# **SUPPLEMENTARY INFORMATION**

## **Supplementary Fig. 1. Correlations between AI–powered and pathologist-derived assessment of PD-L1 expression versus a consensus of pathologists using absolute counts of PD-L1–positive TCs.** Top: Result of frames-based analysis. These frames represent small sections of WSIs. Sections were manually scored by 5 pathologists. A median (consensus) score was obtained and compared with the AI-powered score. Bottom: Comparison of Pearson’s correlation coefficients for AI-powered assessment of PD-L1–positive TCs vs with a consensus of pathologists (blue boxes) and a single pathologist vs a consensus of pathologists (gray boxes). **a** NSCLC (includes samples from CheckMate 017 [*n* = 18], 057 [*n* = 19], and 063 [*n* = 8]). **b** SCCHN (includes a mix of commercial [*n* = 68] and clinical samples from CheckMate 141 [*n* = 18]). **c** MEL (includes samples from CheckMate 067 [*n* = 57] and 238 [*n* = 71]). **d** UC (includes samples from CheckMate 032 [*n* = 32] and 275 [*n* = 24]). *AI* artificial intelligence, *MEL* melanoma, *NSCLC* non-small cell lung cancer, *PD-L1* programmed death ligand 1, *SCCHN* squamous cell carcinoma of the head and neck, *TC* tumor cell, *UC* urothelial carcinoma, WSI, whole slide image.

**a**


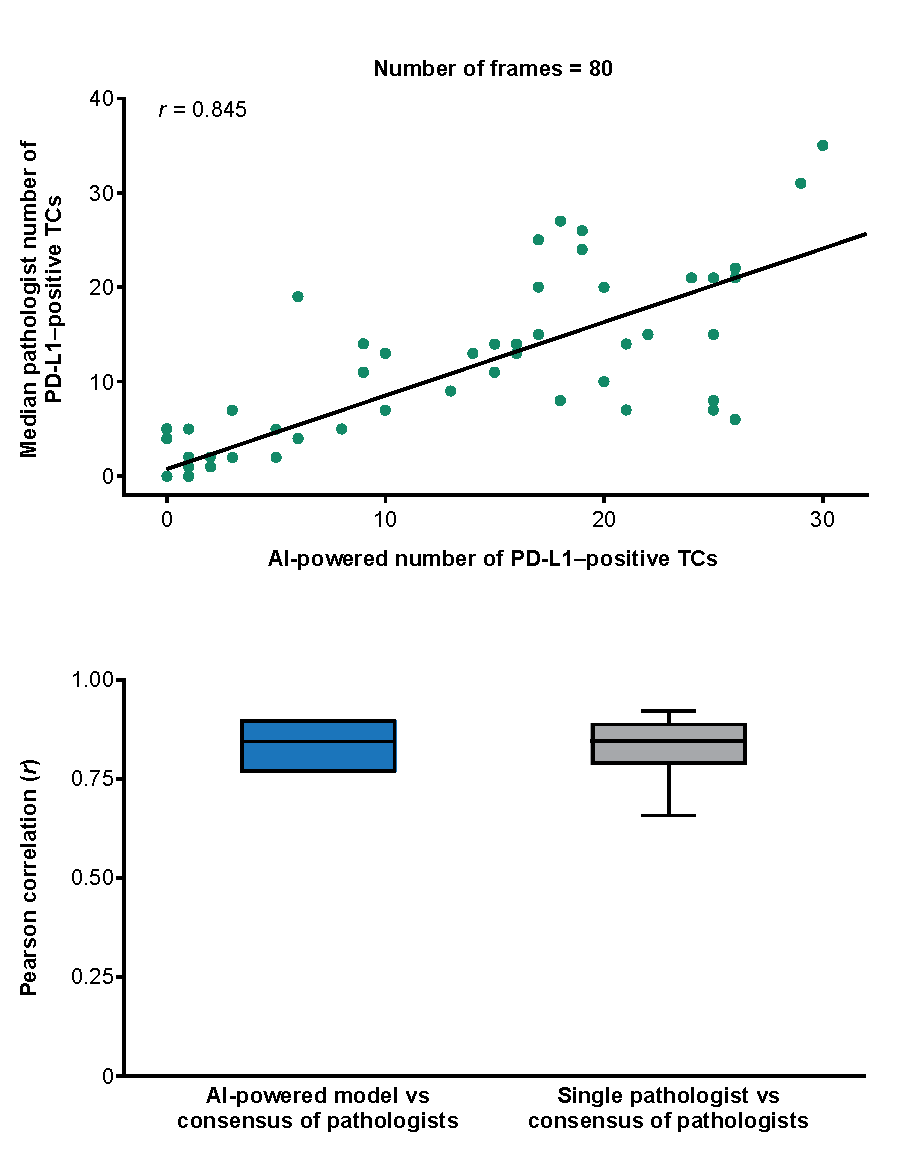


**b**


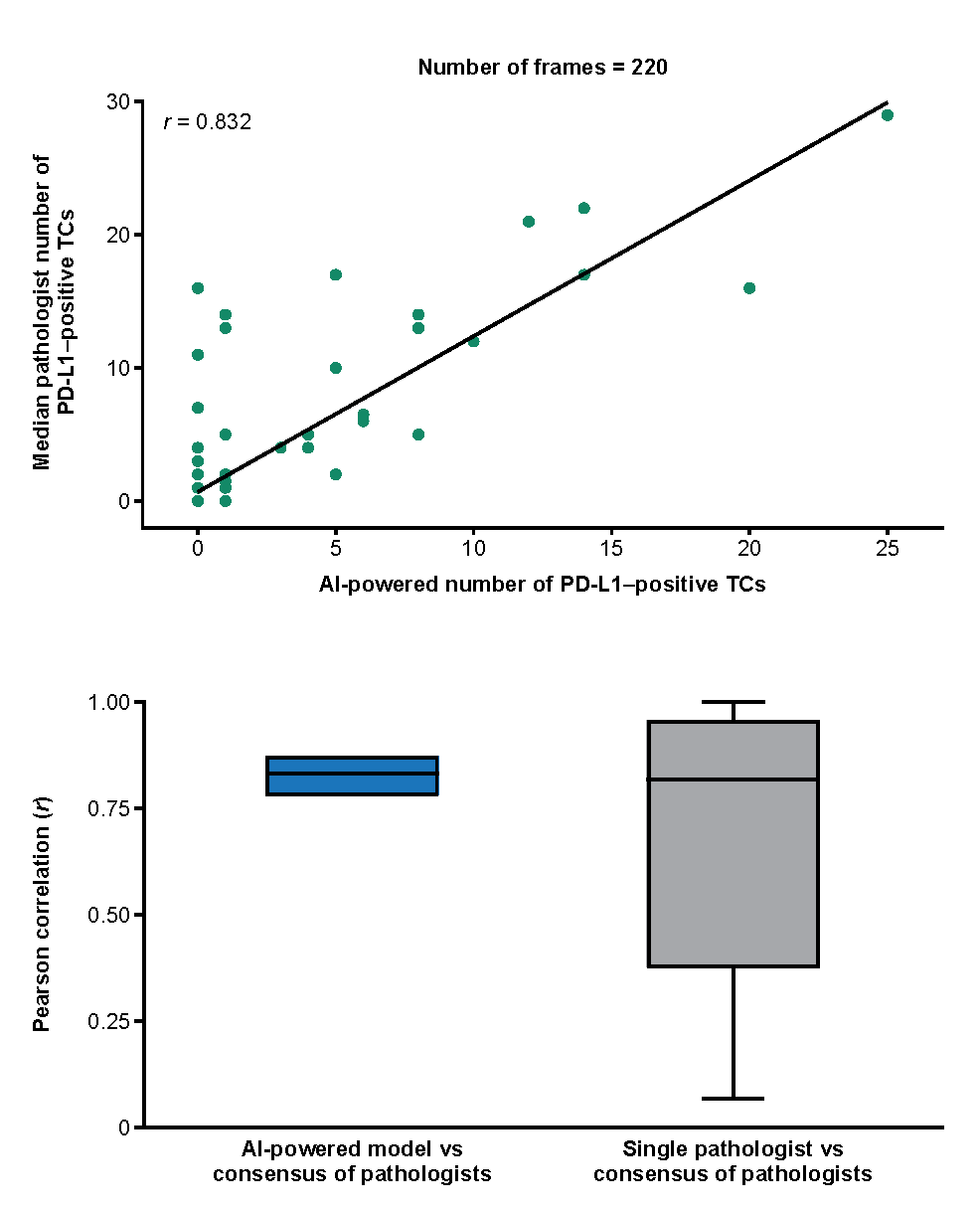


**c**


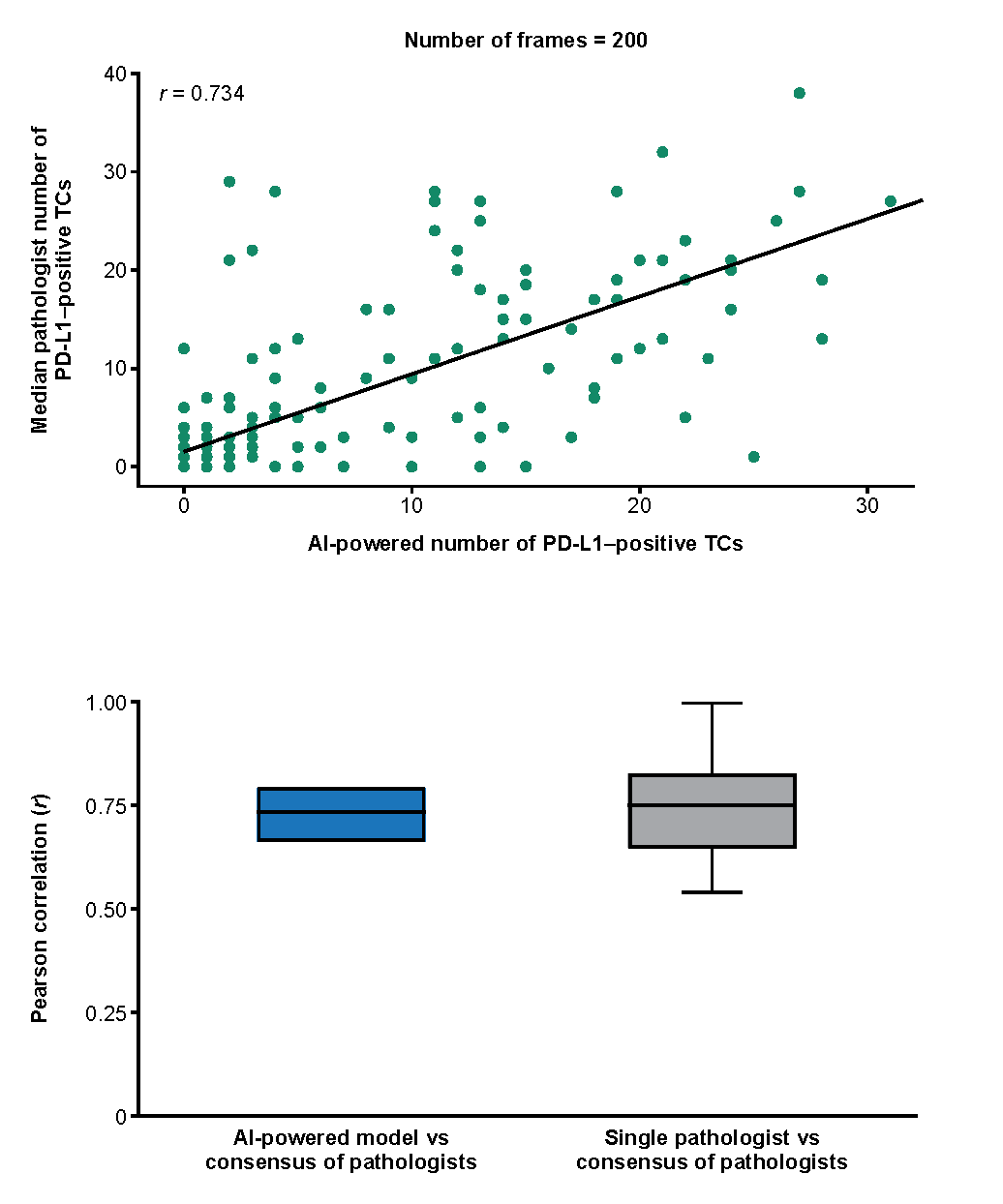


**d**


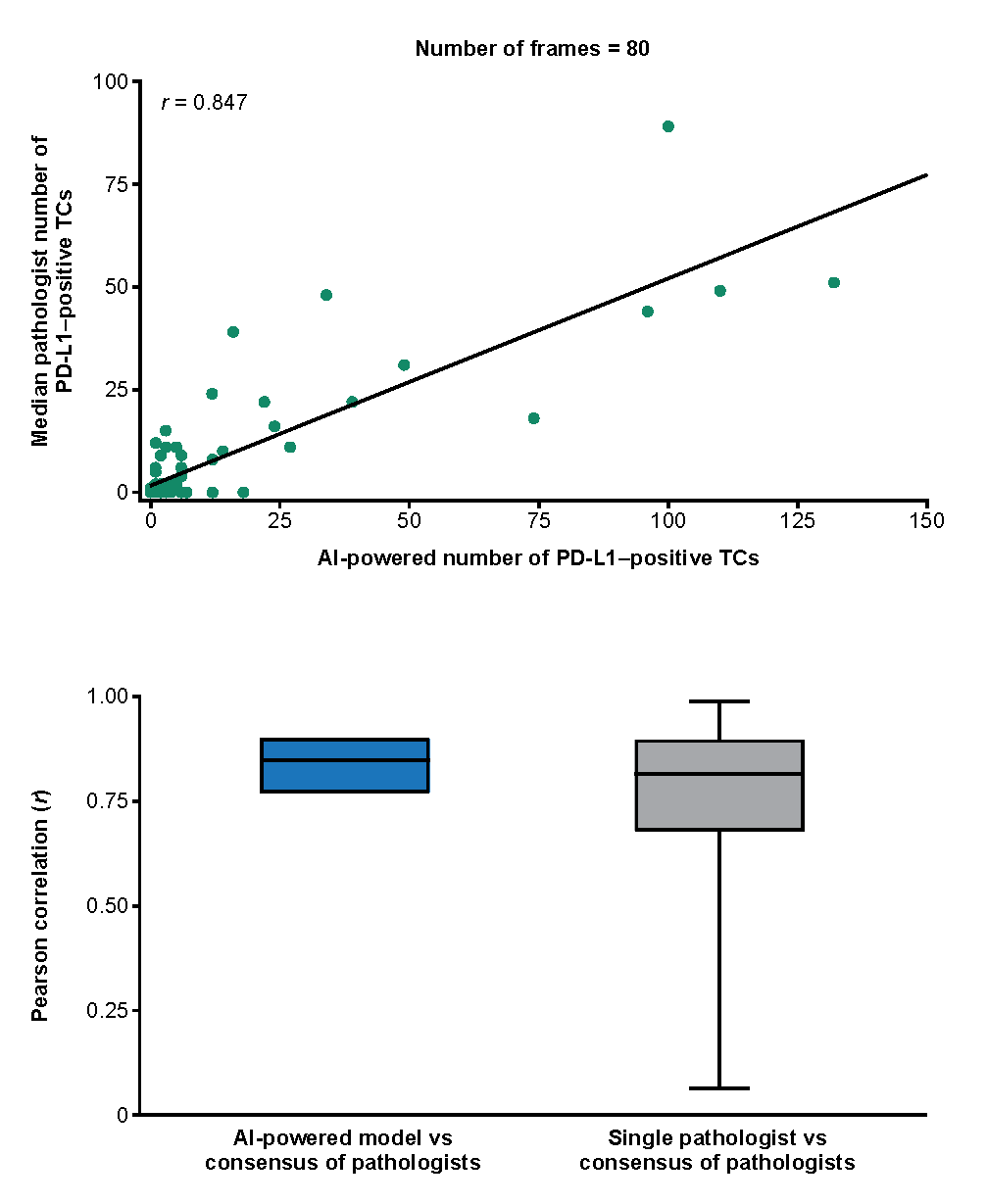


## **Supplementary Fig. 2. Additional analyses of artificial intelligence-powered or manual assessment of PD-L1 expression with survival by trial.** **a** CheckMate 026. **b** CheckMate 057. **c** CheckMate 275. **d** CheckMate 067 (NIVO). **e** CheckMate 067 (NIVO+IPI). **f** CheckMate 238. **g** CheckMate 141. Using the 1% cutoff, there were no patients in CheckMate 057 identified as PD-L1–positive by manual scoring only. *AI* artificial intelligence, *IPI* ipilimumab, *NIVO* nivolumab, *OS* overall survival, *PD-L1* programmed death ligand 1, *PFS* progression-free survival, *RFS* regression-free survival.

**a**

**
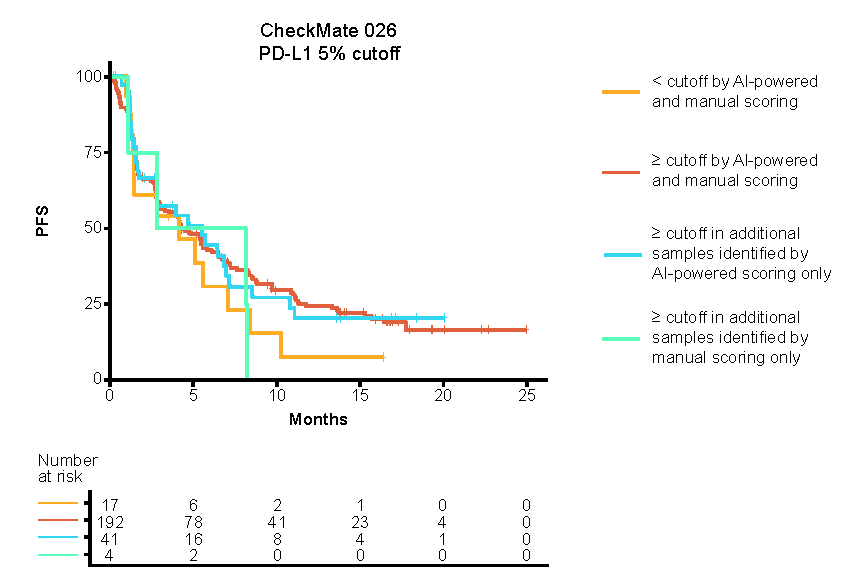
**


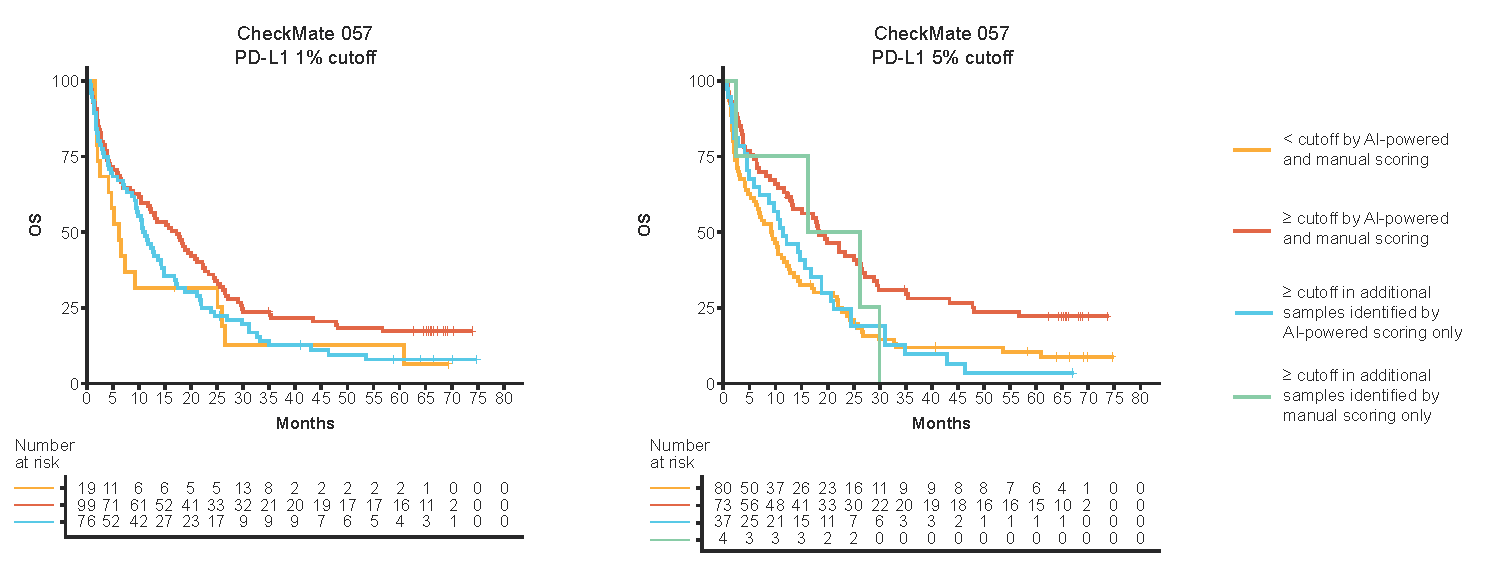
**b**

**
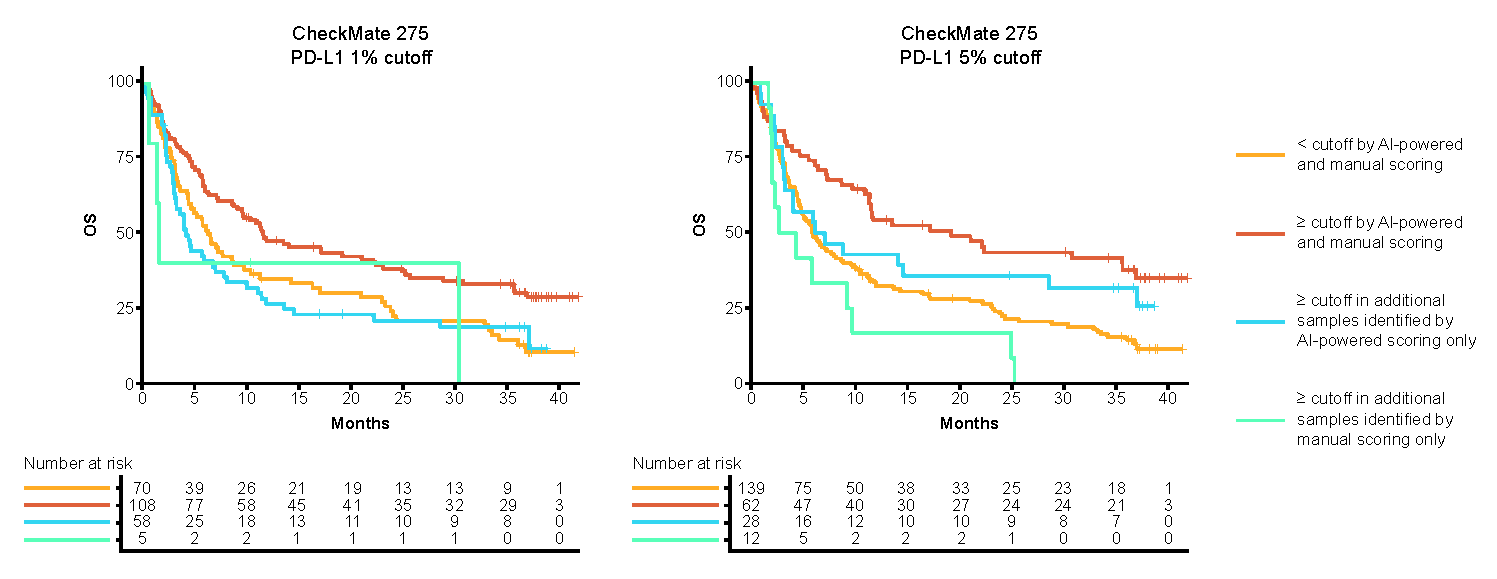
c**


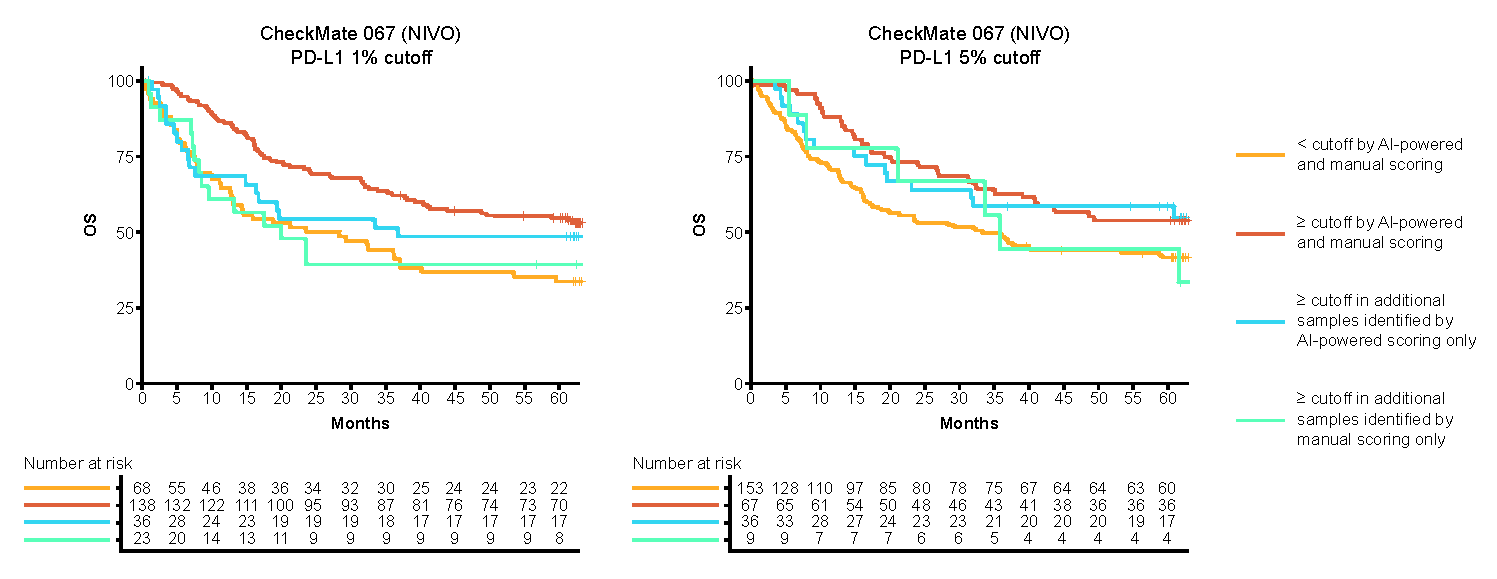
**d**


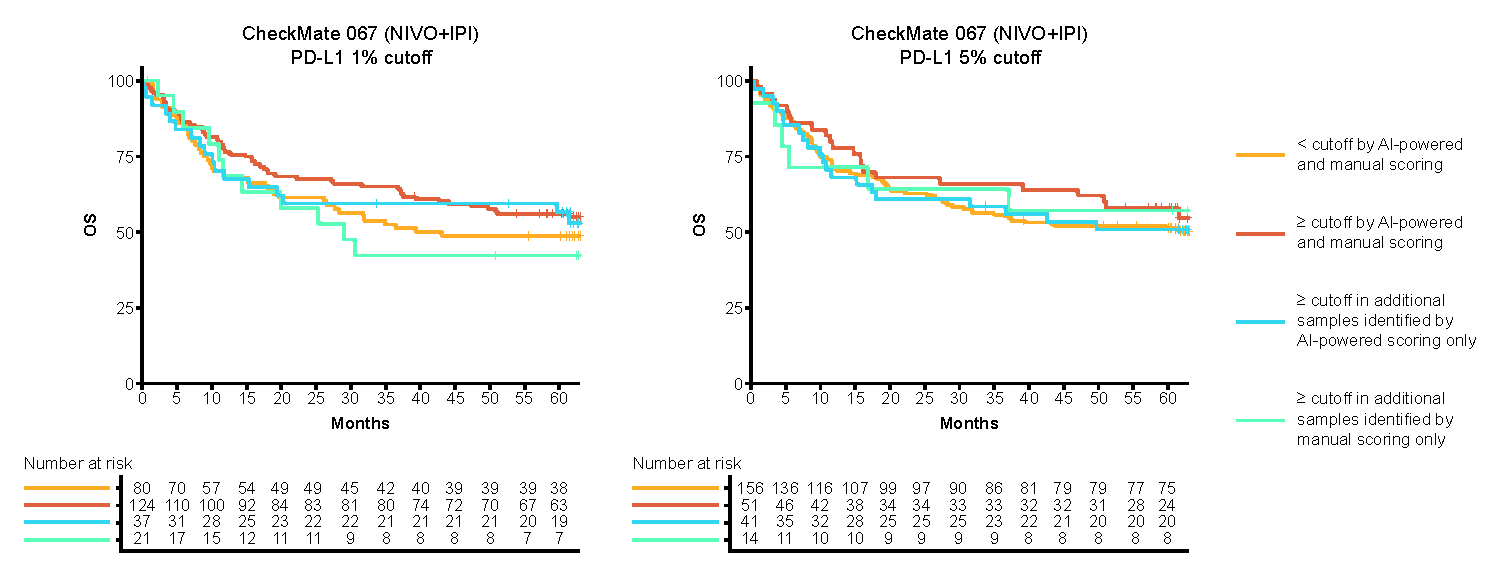
**e**

**f**

**
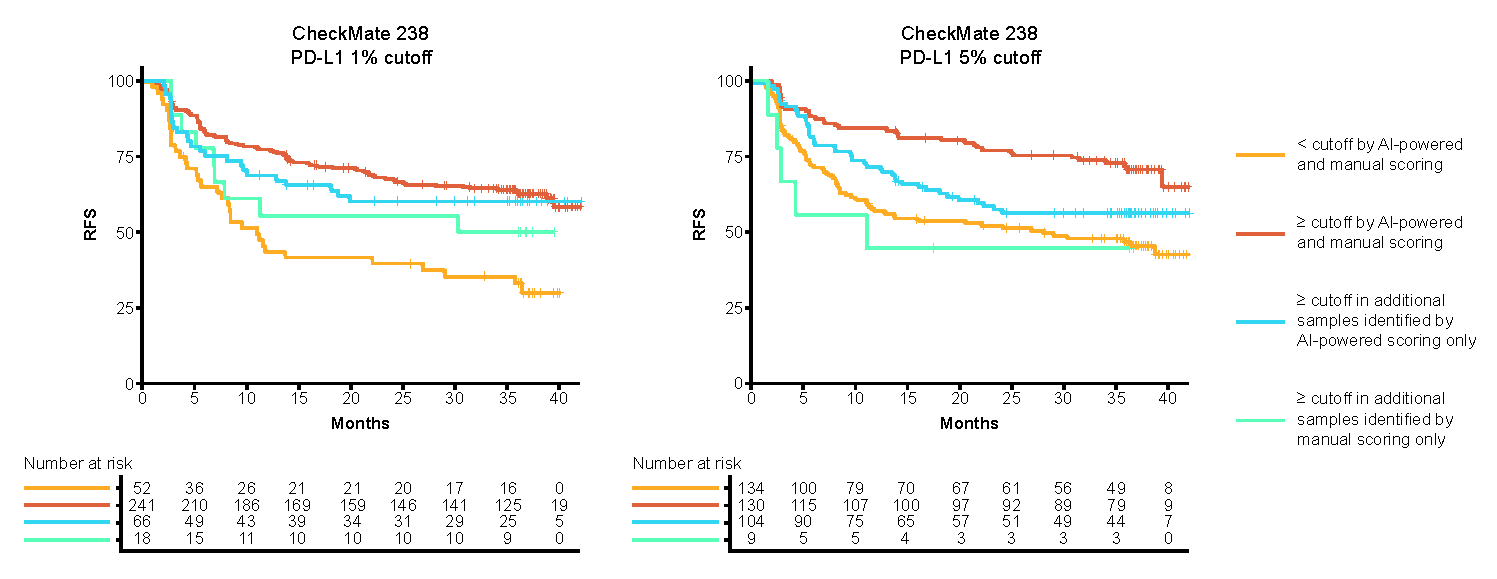
**


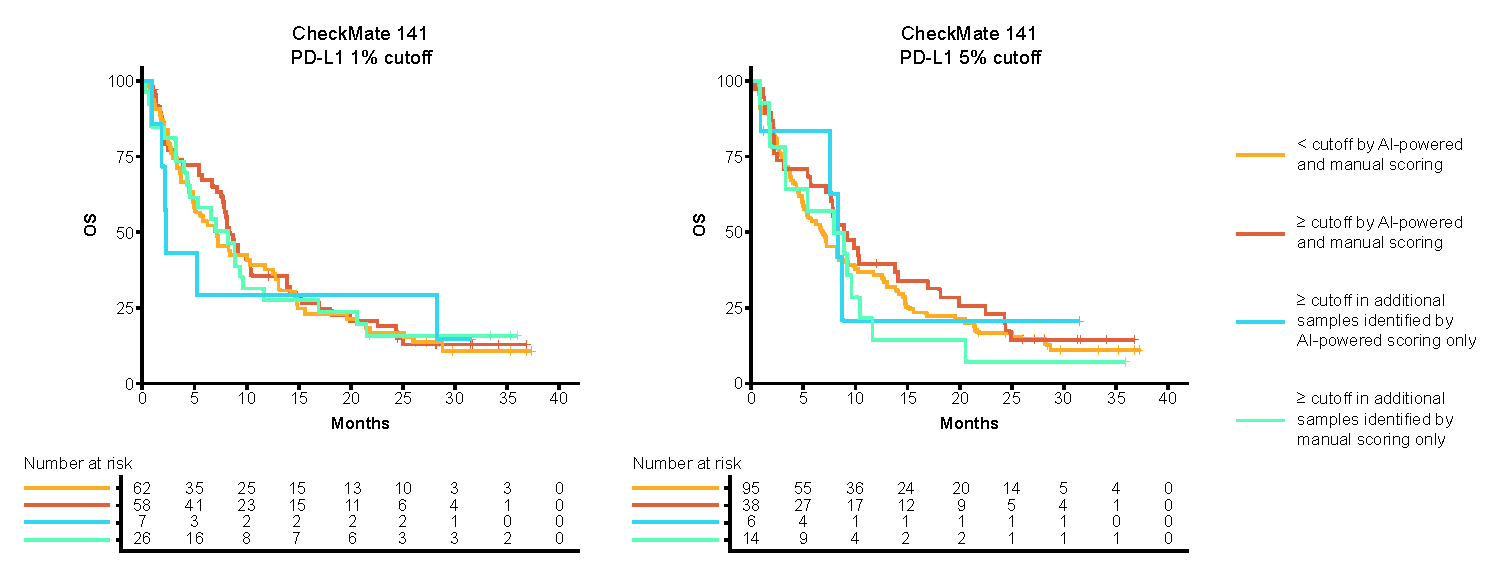
**g**

## **Supplementary Table 1** Additional details on samples used for training of the artificial intelligence–powered algorithm.

| **Tumor Type** | **Origin of slides used for training** | **Number of training slides** | **Number of testing slides** | **Training annotations** | **Frames-based annotations** |
| --- | --- | --- | --- | --- | --- |
| SCCHN | CheckMate 141 and commercial samples | 605 | 88 | 154,483 | 27,947 |
| MEL | CheckMate 067, 238, and commercial samples | 600 | 1540 | 214,162 | 150,238 |
| UC | CheckMate 032[1], 275, and commercial samples | 350 | 87 | 110,000 | 51,595 |
| NSCLC | CheckMate 017[2], 057, 063[3] and commercial samples | 405 | 573 | 99,824 | 46,633 |

*MEL* melanoma, *NSCLC* non-small cell lung cancer, *SCCHN* squamous cell carcinoma of the head and neck, *UC* urothelial carcinoma.

## **SUPPLEMENTARY METHODS**

### **Study designs and treatment**

The study protocols for CheckMate 026 [4], 057 [5], 067 [6], 141 [7], 238 [8], and 275 [9] were approved by the institutional review board at each participating study site. All trials were conducted in accordance with the provisions of the Declaration of Helsinki and Good Clinical Practice guidelines as defined by the International Conference on Harmonisation. All patients provided written informed consent before enrollment. Data and safety monitoring committees provided oversight of safety and efficacy.

### **Clinical assessments**

Tumor response was assessed according to Response Evaluation Criteria in Solid Tumors v1.1 every 6 weeks until week 48 and every 12 weeks thereafter (CheckMate 026) [4], at week 9 and every 6 weeks thereafter until disease progression or unacceptable levels of drug toxicity (CheckMate 057 and 141) [5,7], at 12 weeks, then every 6 weeks for 49 weeks, and then every 12 weeks until progression or discontinuation of treatment (CheckMate 067) [6], and every 8 weeks for 48 weeks and then every 12 weeks until disease progression and treatment discontinuation (CheckMate 275) [9]. In CheckMate 238, patients were assessed for recurrence every 12 weeks for the first 2 years and every 6 months thereafter up to 5 years with recurrence-free survival (RFS) as the primary endpoint[8]. In CheckMate 026, survival was assessed using progression-free survival (PFS) and overall survival (OS) as the primary and secondary endpoints, respectively [4]. In CheckMate 067 and 275 [6,9], survival was assessed using both PFS and OS as co-primary and secondary endpoints, respectively. For the rest of the studies, survival was assessed by OS [5,7].

### **PD-L1 assessment in each clinical trial**

Expression of PD-L1 was assessed centrally using the anti–PD-L1 antibody (clone 28-8) on pretreatment fresh or archival tumor biopsies (Dako IHC 28-8 pharmDx assay).

# **SUPPLEMENTARY REFERENCES**

1 Sharma P, Siefker-Radtke A, de Braud F, Basso U, Calvo E, Bono P, et al. Nivolumab alone and with ipilimumab in previously treated metastatic urothelial carcinoma: CheckMate 032 nivolumab 1 mg/kg plus ipilimumab 3 mg/kg expansion cohort results. *J Clin Oncol.* **37**, 1608-1616(2019)

2 Brahmer J, Reckamp KL, Baas P, Crinò L, Eberhardt WEE, Poddubskaya E, et al. Nivolumab versus docetaxel in advanced squamous-cell non-small-cell lung cancer. *N Engl J Med.* **373**, 123–135(2015)

3 Rizvi NA, Mazieres J, Planchard D, Stinchcombe TE, Dy GK, Antonia SJ, et al. Activity and safety of nivolumab, an anti-PD-1 immune checkpoint inhibitor, for patients with advanced, refractory squamous non-small-cell lung cancer (CheckMate 063): a phase 2, single-arm trial. *Lancet Oncol.* **16**, 257-265(2015)

4 Carbone DP, Reck M, Paz-Ares L, Creelan B, Horn L, Steins M, et al. First-line nivolumab in stage IV or recurrent non-small-cell lung cancer. *N Engl J Med.* **376**, 2415–2426(2017)

5 Borghaei H, Paz-Ares L, Horn L, Spigel DR, Steins M, Ready NE, et al. Nivolumab versus docetaxel in advanced nonsquamous non-small-cell lung cancer. *N Engl J Med.* **373**, 1627–1639(2015)

6 Larkin J, Chiarion-Sileni V, Gonzalez R, Grob JJ, Cowey CL, Lao CD, et al. Combined nivolumab and ipilimumab or monotherapy in previously untreated melanoma. *N Engl J Med.* **373**, 23–34(2015)

7 Ferris RL, Blumenschein Jr G, Fayette J, Guigay J, Colevas AD, Licitra L, et al. Nivolumab for recurrent squamous-cell carcinoma of the head and neck. *N Engl J Med.* **375**, 1856–1867(2016)

8 Weber J, Mandalá M, Del Vecchio M, Gogas HJ, Arance AM, Cowey CL, et al. Adjuvant nivolumab versus ipilimumab in resected stage III or IV melanoma. *N Engl J Med.* **377**, 1824-1835(2017)

9 Sharma P, Retz M, Siefker-Radtke A, Baron A, Necchi A, Bedke J, et al. Nivolumab in metastatic urothelial carcinoma after platinum therapy (CheckMate 275): a multicentre, single-arm, phase 2 trial. *Lancet Oncol.* **18**, 312–322(2017)
